# The clinical dilemma of the persistent idiopathic pneumoperitoneum: A case report

**DOI:** 10.1016/j.ijscr.2019.08.015

**Published:** 2019-08-17

**Authors:** Enda Hannan, Eltaib Saad, Shu Hoashi, Desmond Toomey

**Affiliations:** Department of General Surgery, Regional Hospital Mullingar, Ireland

**Keywords:** Idiopathic, Pneumoperitoneum, Spontaneous, Conservative, Laparotomy, Perforation

## Abstract

**Introduction:**

Idiopathic pneumoperitoneum (IP) is a rare phenomenon which merits special attention. It is defined as free intraperitoneal air which is not caused by viscus perforation and does not require surgical intervention. It is generally considered a diagnosis of exclusion and often poses a genuine diagnostic dilemma.

**Presentation of case:**

We present an unusual case of persistent pneumoperitoneum with no identifiable cause which was found incidentally on a computed tomography (CT) scan in a patient with chronic cough and no prior surgical history. Serial consults revealed no abdominal symptoms or signs. He was managed conservatively and remains asymptomatic despite having a persistent IP on serial radiology.

**Discussion:**

To our knowledge, our case is the first in the literature of an idiopathic pneumoperitoneum that persisted on subsequent radiology and was managed successfully without surgical intervention. This is a highly important case for all practicing general surgeons to learn from as knowledge of this phenomenom may help avoid unnecessary surgical intervention and potential morbidities associated with this.

**Conclusion:**

IP is a diagnosis of exclusion which should only be made after surgical and non-surgical causes have been outruled. In the absence of signs of peritonitis and evidence of gastrointestinal perforation on CT, a conservative approach is warranted, allowing patients to avoid unnecessary surgical intervention.

## Highlights

• Idiopathic pneumoperitoneum is a rare clinical entity that is challenging to identify and manage.
• Unneccessary surgical intervention in idiopathic pneumoperitoneum can lead to significant morbidity and even mortality.
• Conservative treatment with close observation is optimal if no peritonitis or radiological evidence of perforation.
• Contrast-enhanced CT may help exclude gastrointestinal perforation with 86% accuracy and thus help avoid unnecessary surgical intervention.

## 1 Introduction

Idiopathic pneumopertoneum (IP) is a rare clinical entity [[1], [2], [3], [4], [5]]. In over 90% of cases, pneumoperitoneum is due to hollow viscus perforation, usually requiring urgent surgical intervention [1,6]. The remaining 10% of cases are due to non-surgical entities that include various iatrogenic, intra-thoracic, and gynecological causes [[5], [6], [7]]. Rarely, when no cause can be identified, the patient is considered to have an IP [1,2,5]. The pathophysiology of IP is poorly understood and is largely regarded a diagnosis of exclusion [2]. This group of patients require close attention to recognise those who can be safely managed conservatively, thereby avoiding unnecessary surgical intervention [1,8,9].

We present an exceedingly rare case of persistent IP in a 71-year-old patient which was found incidentally on a thoracic CT scan during investigation of chronic cough that has persisted on serial radiology. Our case is unique as we demonstrated a persistent pneumoperitoneum at 4 month interval which was managed conservatively successfully. The following case has been reported in line with the SCARE criteria [10].

## 2 Case presentation

**Fig. 1:**
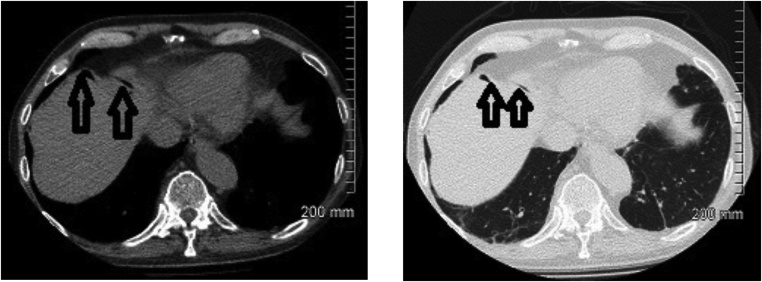
CT thorax showing a right sub-diaphragmatic free air (vertical arrows).

**Fig. 2:**
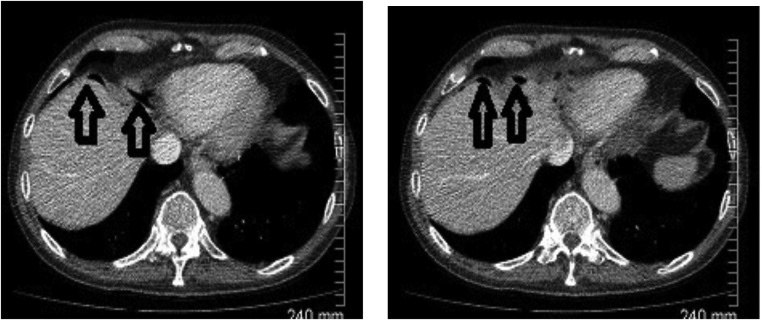
Contrast-enhanced CT abdomen confirming pockets of free intraperitoneal air just anterior to the liver and close to the gastric antrum (vertical arrows).

A 71-year-old Caucasian male presented to the emergency department with productive cough and dyspnoea on a background of recurrent lower respiratory tract infections (LRTI). Of note, he had no prior history of abdominal surgery. He reported being a lifelong non-smoker. His physical examination was unremarkable and his vital signs were all within normal limits. Laboratory investigations revealed a CRP of 102 nmol/L but were otherwise within normal ranges. His chest radiograph demonstrated no gross abnormality. He was initially treated as a presumed LRTI with intravenous antibiotics. A CT thorax demonstrated multiple pockets of sub-diaphragmatic intraperitoneal free air just anterior to the liver and close to the gastric antrum, raising concern for gastric perforation (Fig. 1). Despite this, the patient had no abdominal pain and his abdomen was soft and non-tender on clinical examination. A contrast-enhanced abdominal CT concurred with the above findings of pockets of free intra-abdominal air (Fig. 2). However, there was no other evidence of viscus perforation on the scan, with an unremarkable gastrointestinal tract apart from mild uncomplicated sigmoid diverticulosis. The patient was observed closely with frequent repeat abdominal examinations but remained clinically well without evidence of peritonitis. He was discharged upon resolution of his respiratory symptoms. A follow-up CT scan of the chest, abdomen and pelvis after 4 months showed persistence of the previously noted intraperitoneal free air. All intra-abdominal viscera appeared largely unremarkable once again. The patient remained asymptomatic on serial outpatient consultations.

## 3 Discussion

Pneumoperitoneum is typically thought to represent a surgical emergency. This is with good reason, as over 90% of reported cases are due gastrointestinal perforation [1,6]. Pneumoperitoneum may also be iatrogenic in nature, potentially persisting as long as four weeks post-laparoscopy or laparotomy, or as a complication of gastrointestinal endoscopy [7].

Non-surgical pneumoperitoneum (NSP) is found less frequently [[6], [7], [8]]. Intra-thoracic causes include thoracic trauma (such as barotrauma), post-cardio-pulmonary resuscitation and excessive mechanical ventilation with high peak inspiratory pressures [7,8]. In these cases, pneumoperitoneum is usually a result of raised intra-thoracic pressure, which leads to the leakage of intrathoracic air through microscopic pleural and diaphragmatic defects, and pneumomediastinum often co-exists [2]. In such cases, management of pneumoperitoneum is typically conservative [7,8]. In the female population, gynaecological causes such as sexual intercourse, vaginal douching, vaginal insufflation, and pelvic inflammatory disease, should be considered and are usually a result of anatomical communication between the peritoneal cavity and the fallopian tubes and endometrium [7]. In extremely rare cases, pneumoperitoneum may occur as a result of jacuzzi usage or scuba diving [11,12].

We present a rare case of an incidental finding of a pneumoperitoneum with no identifiable cause that has persisted on radiological follow-up 4 months after it was initially detected. What is unique is our case is the persistence of pneumperitoneum over a period of months in a clinically-well and asymptomatic patient. Only a handful of cases of in the literature described a recurrent pattern of IP, but in these cases, all underwent negative exploratory laparotomies, while ours was successfully managed conservatively [3,5,13]. To our knowledge, our case is the first in the literature of an idiopathic pneumoperitoneum that persisted on subsequent radiology and was managed successfully without surgical intervention. Our case is important as awareness of such a phenomenon may help general surgeons avoid unnecessary surgical interenvtion which may result in significant morbidity or even mortality.

Such a finding certainly poses a significant challenge to the clinician with regards to investigation and management, particularly as the initial concern upon finding a pneumoperitoneum is of an acute gastrointestinal condition requiring urgent surgical intervention [1,8,9]. The patient with the incidentally found pneumoperitoneum frequently undergoes surgical exploration, putting them at risk of various surgical and anaesthetic complications associated with such an approach [3,9]. Van Gelder et al. reported on six patients with spontaneous pneumoperitoneum who underwent negative exploratory laparotomies [4]. With keeping this in mind, it is important, when encountered with pneumoperitoneum, to have an approach for identifying which patients require urgent surgical intervention and which can be managed conservatively.

A detailed history and examination is essential in distinguishing surgical from nonsurgical pneumoperitoneum, thus avoiding unnecessary surgical intervention [4,9,14]. The presence or absence of peritonitis on clinical examination and the underlying cause of pneumoperitoneum should determine whether treatment is surgical or not, as opposed to the mere presence of pneumoperitoneum alone. In cases of NSP without signs of peritonitis, conservative treatment with close observation is indicated [3,4,9,12,14]. With regards to identifying an underlying cause, the first step should be exclusion of gastrointestinal perforation, and this is best achieved by contrast-enhanced CT, which can predict the location of gastrointestinal perforation with 86% accuracy [15].

## 4 Conclusion

IP is a diagnosis of exclusion which should only be made after surgical and non-surgical causes have been outruled. We describe a rare case of persistent IP which was successfully managed conservatively. Our case is unique as the pneumoperitoneum persisted radiologically at a 4 month interval and was successfully managed conservatively. Although pneumoperitoneum is often associated with significant intra-abdominal pathology, in those without clinical signs of peritonitis and without evidence of gastrointestinal perforation on contrast-enhanced abdominal CT, a conservative approach is warranted, thus allowing patients to avoid unnecessary surgical intervention.

## Funding

No funding was sought or received for the purposes of this study.

## Ethical approval

Not applicable. This case report is exempt from ethical approval in our institution.

## Consent

Fully informed written consent was obtained from the patient and documented in the medical notes. No identifying information has been used in this article.

## Author contribution

All authors read and approved the final manuscript.

S Hoashi identified the subject and treated the patient.

D Toomey treated the patient and acted as senior author.

E Saad performed the literature review.

E Hannan wrote the final draft and assisted in the literature review.

## Registration of research studies

This is a case report and does not refer to research involving human studies.

## Guarantor

Mr Enda Hannan.

## Provenance and peer review

Not commissioned, externally peer-reviewed.

## Declaration of Competing Interest

The authors declare no conflict of interests.
